# Editorial: The Marketization of Higher Education: The State of the Union Between the Student as Consumer and the Free Market

**DOI:** 10.3389/fpsyg.2022.932122

**Published:** 2022-07-05

**Authors:** Chris Howard, Carl Senior, Edward J. Stupple, Andrew Corcoran, Yasuhiro Igarashi

**Affiliations:** ^1^Psychology, University of Derby, Derby, United Kingdom; ^2^Aston University, Birmingham, United Kingdom; ^3^University of Derby, Derby, United Kingdom; ^4^University of Nottingham, Nottingham, United Kingdom; ^5^Yamano College of Aesthetics, Hachioji, Japan

**Keywords:** marketization, higher education, quantitative and qualitative, Lacan (Jacques), free market capitalism, student as consumer

There is no doubt that the higher education sector has undergone a seismic shift in its delivery. Large-scale factors from the viral to the geopolitical have shaken the global foundation of universities everywhere. However, despite the storied calls of educational scholars, the university remains at its core a large-scale business (see e.g., Senior et al., 2017), and as any MBA student will readily recite, the aim of a business is to increase the profits of its stakeholders. With this context in mind, the question that was posed to the scholarly community at large was who are the stakeholders and what context do these stakeholders need to operate efficiently? There is no doubt that for the effective development of a free-market structure to be championed there must be due consideration for all stakeholders in the marketplace. Yet this is often something that is ignored by the marketing scholars who tend to focus their efforts on a singular party in any market-based relationship. The seven articles contributing to this Research Topic describe the nature of the free-market context within which a university's main stakeholders function. The articles are diverse in methodology, scope, and perspective on the subject of the marketisation of higher education. As intended, the difference in ideas and findings across the articles reveal a series of contradictions and tensions but also similarities, which offer a wholesome critical engagement and synthesis of the Research Topic.

Taking a student-focused perspective are the articles by Fernandez-Garcia et al. and Itzkovich; they describe how the ephemeral concept of student satisfaction can be used to form a psychological contract between student and university. Both these articles highlight the role grades play in shaping student satisfaction, which is situated within the wider academic environment and clinical practice (Fernandez-Garcia et al.), alongside expectations of who is held responsible (Itzkovich), which echoes the “student as consumer” narrative. In contrast, Xu et al. bring a distinctly institutional perspective that looks to our understanding of transition, government, and effective market behaviors over the last two decades. Senior et al. make a valiant claim that despite the changes that the higher education sector is currently undergoing, institutional administrators must continue to champion their primacy in all aspects of institutional delivery. Overall, this commentary provides a warning for university managers to address the student body not as a collection of alienated individuals but as a functioning community. Bashir et al. continue to develop this message in the context of the post-Covid university that champions a hybrid or blended model between online and on-campus provision to achieve high student outcomes.

The interaction between the social-psychological dimensions of learning, academic achievement, and student complaints is taken up in the explorative article by Bunce. This research helps to contextualize student complaints in the marketized university by bringing to the fore the strength of a student's disciplinary identity, how they approach their studies, and what they achieve. Bunn et al. take a unique and innovative approach that casts a Lacanian psychoanalytic gaze on the “student journey” narrative that is built on the steady acquisition of skills during the course of a degree. The article questions this narrative through five student vignettes that draw upon Lacan's four discourses (master, university, hysteric, and analyst) to demonstrate that the journey is complex, non-linear, and hazardous, and it is shaped not by rational choices but by the never-ending quest to fill the lack in desire. Importantly, this analysis highlights that the experiences of students within the marketized university cannot be taken in isolation but should be understood as being produced by wider social, cultural, and political forces. The seven articles nonetheless demonstrate the breadth and depth of work on the marketization of higher education. They provide an evidence base for educators to build upon and use in practice but also a set of diverse, innovative, and rigorous methodological tools to work with to further the field.

## Author Contributions

All authors listed have made a substantial, direct, and intellectual contribution to the work and approved it for publication.

## Conflict of Interest

The authors declare that the research was conducted in the absence of any commercial or financial relationships that could be construed as a potential conflict of interest.

## Publisher's Note

All claims expressed in this article are solely those of the author and do not necessarily represent those of their affiliated organizations, or those of the publisher, the editors and the reviewers. Any product that may be evaluated in this article, or claim that may be made by its manufacturer, is not guaranteed or endorsed by the publisher.

## References

[B1] Senior C. Moores E. Burgess A. P. (2017) “I Can't Get No Satisfaction”: measuring student satisfaction in the age of a consumerist higher education. Front. Psychol. 8, 980. 10.3389/fpsyg.2017.00980 PMC5462985 28642730

